# ‘A fiction author can do anything, we’re bound by the facts’: The risks and opportunities of taking advantage of cognitive biases in storytelling for science communication

**DOI:** 10.1177/09636625251387445

**Published:** 2025-11-22

**Authors:** Hannah Little, Juliet Dunstone

**Affiliations:** University of Liverpool, UK; University of Liverpool, UK; University of Stirling, UK

**Keywords:** cognitive science, cultural evolution, science communication, storytelling

## Abstract

Storytelling is a growing topic in science communication research, highlighting the importance of learning from existing storytelling research from other disciplines. Storytelling research in cultural evolution has identified a number of cognitive biases in how we transmit information: stories are remembered and passed on more faithfully when they contain social and survival information, negative information or counterintuitive information. In this article, we review this cultural evolution literature and present findings from a set of interviews with science communication professionals. We asked science communicators about the potential benefits and risks that may come about when using cognitive biases within science communication storytelling. Science communicators reported already using some cognitive biases in their practice. Participants also expressed concerns about some tactics that might contradict objectives of science communication, threaten the integrity of science and science communication and risk the welfare of audiences. We map the benefits and risks reported using a thematic analysis.

## Storytelling in science communication

Humans have always used stories to understand the world around us, from folktales to news stories, and from movies to the gossip we collect in the local pub. Science communicators have long been trying to capitalise on this most human instinct to hear and pass on the information we hear in the form of stories.

For science communication, stories enable us to foreground relevance, emotion and engagement, which can be used to persuade audiences or make information stick with people. Many people already get their science content through the mass media, which has a bias towards communicating through narratives (Dahlstrom, 2014), and many science communicators use storytelling in communication through video, stage shows or writing. As a result, research centring on the use of stories within science communication has exploded in recent years, inspiring dedicated books on the subject (e.g. Olson, 2018) and special issues and edited books (e.g. Coren and Wang, 2024; Joubert et al., 2019).

While much of the science communication literature explores how communication professionals can use stories, it is largely missing engagement with literature from other disciplines about what makes stories memorable. Literature from the field of cultural evolution and cognitive science has been investigating for years what makes some stories stick with us while others are lost, and what makes us remember information from the stories we hear (see Berl et al., 2021 for a summary).

In this article, we first review the empirical literature from the cultural evolution of stories, before engaging with science communication professionals in an interview study. We ask them how they use stories, whether they can benefit from the evidence reviewed in this article about cognitive biases for certain types of narratives and what issues the cognitive biases reviewed might pose for their science communication practice. The article is not intended to justify the use of storytelling as a science communication practice, as this is done elsewhere. Instead, it aims to analyse considerations from science communicators already using storytelling.

## Cognitive biases in storytelling

The literature from the fields of psychology, anthropology, cultural evolution and cognitive science indicates that stories are remembered and passed on when they have features that take advantage of our cognitive biases. For instance, a cognitive bias towards social information. These cognitive biases cause humans to attend to and remember certain types of information more than others, rather than other types of bias which may influence our understanding of information (e.g. in ways that might bias audiences against truth or certain demographics) and this manifests in the types of stories that we remember and pass on.

Investigations into what makes stories memorable first arose when scholars noticed similarities in the folktales of different cultures across the world. Were these similarities down to some shared origin, or due to humans simply being biased towards certain themes and structures within stories?

Addressing these questions is difficult because records of folktales predate written records. These are oral traditions, so how would we know what these stories originally looked like, how they changed over time and what stories were lost when no one retold them?

Bartlett (1920) sought to investigate what makes some stories and folktales persist in our collective memories, while others are lost. Because he could not access the stories forgotten from our oral traditions, his work utilised the transmission chain method, similar to the childhood game ‘telephone’, in which speech is whispered from person to person (Bartlett, 1932). In these experiments, Bartlett gave someone a story which they were then asked to recall from memory. Their recollection was given to a new participant to read and recall and so on. Through this process, we can see what information ‘survives’ after passing through several minds. Instead of the survival of the fittest in biological evolution, in cultural evolution we have survival of the memorable (Barrett and Nyhof, 2001).

Bartlett used these transmission chain methods to demonstrate that traditional folk tales were more memorable than other types of text, such as newspaper reports or scientific texts (Bartlett, 1932). These results are perhaps not surprising: folk tales have adapted to the minds of humans after being orally passed on for millenia. However, Bartlett did not explore more nuanced conditions of these experiments that would have allowed identification of exactly what it is about folk tales that makes them so memorable.

Since 2006, more recent studies, discussed below, have sought to find specific aspects of stories that make them more memorable. This article will review evidence for cognitive biases in stories with the following:

1. Social information
2. Counterintuitive information
3. Negative information
4. Survival information

Using storytelling tactics that appeal to these cognitive biases does not necessarily make our communication biased (e.g. against truth or certain demographics) – some stories about science can, by their nature, appeal to our cognitive biases. For example, a surprising scientific result can appeal to our cognitive bias for counterintuitive information without being manipulated in any way, and a scientific finding from medicine that aids survival need not misrepresent the science to have a survival frame. While misinformation and conspiracy theories can misinterpret science to force counterintuitive or survival narratives (Cassam, 2023), we are not condoning the manipulation of science to communicate using cognitive biases.

The cognitive biases discussed in this article are similar to ‘news values’ in journalism, which dictate what makes news stories more likely to get picked up (Harcup and O’Neill, 2017). However, the evidence behind news values lies in the prevalence in how often certain types of stories appear in the news media (Harcup and O’Neill, 2017), rather than in how attractive or memorable these stories are to audiences. In modern media, though, story choice will be heavily informed by analytics for audience numbers.

While much narrative science communication happens in the context of journalistic writing, it is also important to understand how these cognitive biases play out within transmission of stories from person to person. Most information we process every day comes from our peers. The average person will speak 15,000 words a day (Mehl et al., 2007), and in a corpus study, Dessalles (2008) found that 26% of words we use in everyday conversation are used to tell stories. Higher estimates have storytelling making up 40% of our spontaneous conversational language (Eggins and Slade, 2004). If some of those stories could be about science, it could hold great power for science communication.

### Social information bias

Social information bias was the first cognitive bias in storytelling to be empirically tested using the transmission chain, or ‘iterated learning’ method. Social information is defined as information concerning interactions and relationships between a number of people (Mesoudi et al., 2006). This cognitive bias is assumed to exist as a result of the Social Brain Hypothesis (Dunbar, 1998), that our brains evolved primarily to deal with complex social problems, or Social Gossip Theory (Dunbar, 1993), that human language evolved as a means to maintain social cohesion in the large groups.

To test whether this cognitive bias influences how we remember stories, Mesoudi et al. (2006) designed an experiment with four conditions. Participants were given one of four stories with different amounts of social information in them. Either a scientific story with no human characters, where features of a physical environment interacted and influenced each other (e.g. global warming creating forest fires), or a story with one human character who interacts with the physical environment, an everyday story with more than one human character interacting (e.g. someone getting lost and getting directions) and a story with more than one human character interacting in an intense way (e.g. interactions that might be considered extreme or scandalous). The study found that stories with social interactions between more than one human character were remembered significantly more accurately than stories with equivalent amounts of non-social information with one character or fewer. Making the interactions between characters scandalous did not make them more memorable than more everyday interactions, and having one human character was not enough to make a story more memorable than the story with no human characters. This cognitive bias has also been found in other experimental work (e.g. Berl et al., 2021; Stubbersfield et al., 2015).

Social information bias is similar to the Soap Opera Effect (Owens et al., 1979), in which stories are remembered more accurately when the audience has beliefs about a characters’ motives. In an experimental study, Owens et al. (1979) showed that when participants were told about what a character was worrying about, they recalled events in a story more accurately than if they were given no such information.

### Counterintuitive information bias

Counterintuitive information bias was originally outlined by Boyer (1994) as the theory of the transmission of counterintuitive ideas. Counterintuitiveness can be understood as information contradicting what is intuitive. In science communication, intuitive concepts might be called ‘folk biology’ or ‘folk physics’ (Stubbersfield and Tehrani, 2013) and are thought to be cross-cultural (Barrett, 2008). When a story or scientific findings violate these intuitions, this can make transmission of a story more successful (Barrett and Nyhof, 2001; Berl et al., 2021; Boyer, 1994; Boyer and Ramble, 2001; Norenzayan et al., 2006; Stubbersfield and Tehrani, 2013), which is explained by the counterintuitive elements being more salient than intuitive information (Berl et al., 2021). However, research has also established that stories being completely counterintuitive can make them too complex to be understood (Boyer and Ramble, 2016), which can be counterproductive for a story’s transmissibility. A story must strike a balance between non-complex, everyday intuitive events and a small number of counterintuitive elements. This is called the minimally counterintuitive bias (Boyer, 1994).

Counterintuitive information bias is similar to the news value of surprise. News stories are more likely to be covered that are surprising or have an element of contrast (Harcup and O’Neill, 2017). The stories we tell one another on a daily basis often have an element of unexpected or unusual events (Dessalles, 2018). Dessalles (2007) posits that communicating surprising or unexpected events has influenced our evolution: humans gain status through reporting stories of successfully navigating unexpected situations. Upala et al. (2007) suggest something similar, that the reason we favour counterintuitive information is that it is evolutionary advantageous to remember events that violate expectations that may be dangerous.

In science communication, an unexpected or counterintuitive result is more likely to get picked up in the press because of news values (Badenschier and Wormer, 2011). However, this cognitive bias towards counterintuitive information can result in publication bias, as studies with positive results which may go against our expectations are less likely to be true, but more likely to be published (Tincani and Travers, 2019). Dessalles (2018) found that within our everyday conversations, reliability comes second to our need for our stories to be unexpected, possibly creating a risk for the reliability of science communication seeking to monopolise on this cognitive bias without care.

### Negative information bias

Negative information bias is characterised by humans disproportionately paying attention to negatively valenced information. Using a cultural transmission experiment, Bebbington et al. (2017) found that more information was recalled from stories containing negative events than those with positive events. Furthermore, they found that stories with ambiguous events, where it wasn’t clear whether the events had an either positive or negative effect, became negatively valenced over the course of transmission chains. In a similar study, Berl et al. (2021) found that people remembered more information from stories with negative emotional information than stories using the other cognitive biases discussed in this article.

The cognitive bias for negative emotional information has also been hypothesised to have an evolutionary explanation (Baumeister et al., 2001). Paying attention to information that triggers fear, disgust or anger ensures we attend to stories about things that may be dangerous to us (Al-Shawaf et al., 2016). This may also explain why negative information is detected faster than positive information (Dijksterhuis and Aarts, 2003), and why negative feedback has a greater effect on us than equally intense positive feedback (see Baumeister et al., 2001 for a review).

Negative information bias is similar to the news value of bad news. News stories with conflict or tragedy are more likely to be covered by the news media (Harcup and O’Neill, 2017). However, good news stories, such as miracle cures or people being rescued from peril, will also be picked up. McIntyre and Gibson (2016) argue that adding a positive ‘silver lining’ to bad news stories can still attract audiences, without leaving them with negative emotions.

### Survival information bias

Survival information bias is very much linked to the negative information bias. We attend to information that will aid our survival, whether threats or information that can help us overcome threats. For example, for science communicators, survival information might be about avoiding or overcoming life-threatening health conditions, or preventing existential threats to humanity, such as climate change.

Using a cultural transmission experiment, Stubbersfield et al. (2015) found that stories that contained survival information were recalled more accurately than stories without. However, this effect was not as strong as the effect of stories with social information (see above), or stories that had both social and survival information within them. Berl et al. (2021) found similar results in a cultural transmission experiment, finding a clear bias in remembering survival information, but this was not as strong as social or negative information.

Survival information bias also works with words or even pictures without the surrounding story. Nairne et al. (2008) found that words that had connotations of survival were recalled more successfully when the participants imagined they were stranded without the means to survive. This act of imagining may be a reasonable proxy for being caught up in a story. Otgaar et al. (2010) found the same improved recall of survival-themed material, but with pictures instead of words.

## Risks and opportunities for science communicators

The evidence reviewed above supports that the cognitive biases mentioned make stories more memorable, suggesting people are attending to information which takes advantage of these cognitive biases, which may be useful for science communication. However, the evidence reviewed does not speak of other effects these cognitive biases will have on audiences. Objectives in science communication are not usually to simply give information that is remembered and passed on accurately. Information should also be trusted and useful for the recipient, whether in terms of understanding its links to policy, society or individual behaviours (Besley et al., 2018). Therefore, for science communication, understanding the cognitive biases associated with successful storytelling may not be enough to improve science communication practice. For example, survival information could be applicable in a range of science communication contexts, for example, the dangers of a pandemic or extreme weather events. However, feelings that dangers may be too large to be tackled, as often happens with issues in climate change, may result in denial or fatalism.

The aim of the present study is to establish potential applications or issues with implementing cognitive biases within storytelling for science communication. The literature from cultural evolution and cognitive science is rarely cited within the science communication literature, if at all. The novelty of this study therefore lies first, in introducing science communication professionals to concepts from cultural evolution literature, and second, in establishing how this literature might be useful to science communicators and where there might be risks in using it without more targeted considerations from practice and theory. We have established the following four research questions:

1. Are science communicators already taking advantage of cognitive biases within their storytelling practice?
2. What opportunities might cognitive biases offer science communicators?
3. What risks might cognitive biases create for science communicators?
4. Do different objectives and modalities for science communication affect whether or how cognitive biases might be used?

We have selected four cognitive biases to interrogate in the context of applied science communication, those reviewed above. We briefed science communication practitioners on each cognitive bias before asking them whether it might be beneficial or problematic for their objectives, audiences and media. The larger aim of this study is to increase critical engagement among science communicators, both by raising awareness of empirical findings from cognitive science and encouraging reflection on whether science communication objectives are best served by taking advantage of cognitive biases.

## Methods

We used semi-structured interviews to speak of science communication professionals about the risks and opportunities they predict from implementing knowledge of four cognitive biases in their science communication and storytelling practice.

Interviews provide direct access to experiences, opinions and insights of the participants (Tong et al., 2007). Semi-structured interviews are useful to prompt detailed and open discussion to gain a depth of information that covers all areas and allow issues and ideas to be drawn out throughout the interview.

Interviews were conducted online so we could remain as flexible as possible with the timing of interviews and the location of participants. We briefed participants on the context of the work, explaining that cognitive biases can help improve memorability of stories, but differentiating that from other possible objectives within science communication. Participants were briefed on the four cognitive biases presented in this article: information that is social, survival oriented, negative or counterintuitive. For each cognitive bias we gave a definition and asked participants the following:

1. Do you think knowing about this cognitive bias might be beneficial to your science communication practice in some way?
2. Do you foresee any potential problems with framing findings in your science communication to use this cognitive bias?

Participants were able to ask for examples of the cognitive bias if they were unsure about its definition or possible applications.

We also asked participants about their job, how they use storytelling in their work, the audiences they typically communicate with, how long they have worked in science communication and whether they have any qualifications or formal training in science or science communication.

### Participants

We interviewed 19 science communication professionals. Participants were recruited using a specialist science communication mailing list based in the United Kingdom, callouts on social media and snowball sampling. Recruitment advertisements specified that we were looking for in individuals working in the public communication of science, including people working as science journalists, museum professionals, public engagement professionals, including those who provide training, broadcasters, freelancers or informal science educators. We also specified that participants should use storytelling in their practice, but allowed participants to define their use of storytelling themselves as part of our study was understanding how storytelling is used, rather than imposing a definition.

Each participant who expressed interest was vetted via an email asking them about their role to ensure that they met our definition of a science communicator and asking how they used storytelling within their science communication. No participants were rejected because of this vetting.

Participants came from a diverse range of sectors within science communication with a diverse set of expertise and backgrounds. Participants self-defined as science communicators, with responsibilities including journalism and writing for web (N = 6), filmmaking (N = 3), producing digital content (N = 4), presenting stage shows (N = 5), training other science communicators (N = 3) and consultancy (N = 3). Nearly all participants mentioned engaging with more than one of these responsibilities as a part of their work, which is why the above values do not add up to 19.

Participants also differed in their level of science training. All participants except two (N = 17) had completed at least one degree in science (defined broadly as including STEM subjects and social sciences). We also asked whether participants had any formal science communication training; five of them had master’s degrees specifically in science communication. Of those who didn’t, nearly all expressed that they had training in the form of short courses as part of their professional development.

Participants also worked with a diverse range of public audiences, including children (N = 11) and adults (N = 12). Some participants reported working with both adult and younger audiences, sometimes as a part of venues that cater to different audiences at different times (e.g. science centres and museums) or as a part of different projects.

### Coding

Interview transcripts were coded using thematic analysis (Clarke and Braun, 2021). Transcripts were coded once using a deductive approach to code the risks and opportunities science communicators reported for each cognitive bias in relation to their own work. Transcripts were then coded inductively for common themes that emerged across cognitive biases. A secondary analysis was performed by the co-author to ensure themes adequately represented the original data.

## Results

We first present a summary of how participants reported using stories in their communication, then the opportunities and risks they see for each cognitive bias and then cover common themes across the different cognitive biases.

### Storytelling in science communication

Participants’ use of storytelling differed in context, content and media. Participants used stories in stage shows, written articles and online video. They scripted stories to form videos, stage shows or written pieces or interviewed scientists asking them to share their own stories. Some reported using stories in more unconventional ways. For example, structuring a workshop or a museum as a story. One participant said: ‘I always want to work out my workshops in terms of a beginning, middle and end’, while another said: ‘the entire museum is one story, and you follow the thread through gallery after gallery’.

Participants said that stories were a way to make information more engaging, resonate with people, provide a context for the science and make people care about information. Participants believed in the power of stories saying:

You couldn’t have comms without a good story; the best way to talk about science is to tell it as a story; if you don’t use stories, then you’ll lose people; storytelling is absolutely central to my conception of scientific communication.

Participants reported having practical restrictions, such as the amount of time or resources they have to craft stories, and they mentioned that storytelling was easier in some communication media than others. They specifically mentioned that storytelling was easier with longer-form media: ‘If I’m writing a long form, maybe I can write more of the story’; ‘We have longer videos. . . and these are a lot more narrative driven and have a storytelling element to them’. This potentially creates problems for science communicators who work with social media, where content often needs to be shorter.

Some communicators expressed that they occasionally experienced resistance when implementing storytelling techniques from institutions or colleagues they work with, especially when storytelling is inconsistent with a more traditional approach. There were also some difficulties expressed by people who do interview-based work, sharing the stories of others. One participant noted that it was difficult to get scientists specifically to tell stories:

Getting scientists to the point where they actually use storytelling in science anyway is a little bit of a struggle, because they are a bit like ‘ohh, it’s that’s just airy-fairy humanity stuff’, so getting them to that step of storytelling is kind of a little bit difficult.

### Considerations for social information bias

#### Opportunities

Not all participants used science stories that were social, though many reported a willingness to try it out. Some did have experience in using social elements:

If I had to pick out one kind of element of storytelling that we’re really keen on, it is that human element; if you can give an example of a person, if you can have a first-hand testimony, people will connect more with the story and will remember it.

Communicators felt that bringing in social information can ‘humanise’ the science, especially in fields that are more removed from human stories: ‘I do think we dehumanise science and mathematics all too often [. . .] It doesn’t tend to be about the people involved and I think that is a great tragedy’.

Some felt that having diverse characters can make science seem relatable to an audience: ‘When you include [a range of characters] you can’t dismiss that whole story as being about that type of person’.

Participants observed that making stories more social could counter ideas of science being a solo endeavour:

We have a lean towards writing about teams of research and researchers instead of the superstar researcher now. It’s reflective of how science actually works; science is a very complex thing in terms of the number of humans involved in it.

Although some worried that emphasising some characters could overemphasise their role in the science:

You can’t give too much credit to one side because if they hadn’t been for the early work of so and so, this couldn’t have happened. And if you don’t credit them then it’s kind of given all the credit to the new groundbreaking research.

#### Risks

Participants observed that making science ‘social’ was difficult in the context of communication about maths or sciences with less relevance to humans or society. One participant said: ‘The only problems that I have run into is push back from some people in the mathematical community that I am focusing too much on human stories’. Some worried that making stories social might detract from the science being objective: ‘If you get too involved in the social side of things and it’s people’s opinions sometimes rather than facts’.

Others worried that having characters created a risk in terms of not being able to represent diverse audiences:

We often deal with very diverse audiences and so a story that has characters and therefore represents a version of a world, however you’ve built it, is it going to represent the background that I have, or whoever is telling that story? And potentially other people can’t see themselves in it.

For science communicators who work with social media, there was a feeling that social information bias may make content longer:

Everyone wants things to be shorter, so there’s a kind of temptation to not introduce additional characters; the length will increase because you have more people, more characters in play, and when you’re trying to keep things [short] that’s really hard to do when you have more than one person in play.

There were also issues with the communicators not having time to collect materials:

We have a lack of time to have this social interaction or social perspective. It means that we have to talk to people, and sometimes we don’t have much chance to talk to many people; it’s taking the time to mould it a little bit more which I guess sometimes you might not have.

Or they might have issues with representing more than one character in a one-person stage show: ‘I guess the problem would come if you, how do you do it when you’re doing a solo presentation?’

### Considerations for counterintuitive information bias

#### Opportunities

Science communicators generally saw counterintuitive information as something integral to storytelling: ‘I think that’s probably quite an inherent thing that people do in any story’; ‘That’s literally a narrative structure. We expected ’X’ thing to happen and it didn’t’. Participants saw surprise as being a powerful tool:

I always would have assumed that surprise kind of is a pretty powerful factor; surprise is one of the best tools that we have at our disposal; the bigger the gap between what you were expecting and what actually happened, the bigger the satisfaction once you get it. Once the penny drops.

For science communicators who extract stories from others, counterintuitive information was something they tried to tease out: ‘A question I always ask researchers is ‘what surprised you about the thing in question?’’

Counterintuitive information was seen as an opportunity to engage the audience as scientific thinkers:

Setting it up in the show, ‘what do you think is gonna happen?’, will engage people first. And that means there’s more chance of either they got it right and they feel proud of themselves, or they didn’t get it right and they feel puzzled. If you just show them without that setup, I think you’re losing an opportunity there.

Participants felt counterintuitive stories would be ‘particularly useful on social media and things like that where people like kind of shocking things’, or in news stories:

News is about things that are new, or shocking, dramatic. Those are usually the news that you will easily pick and that we know that people read or at least click on these kind of stories that have a dramatic ending.

Some communicators saw counterintuitive elements as a way to prolong engagement and encourage audiences to keep engaging:

So having something that challenges you or challenges your natural thoughts or your previously held thoughts I think would be one way to encourage people to move into the second level of text; you want people to stay and so you don’t reveal everything all at once. Sometimes it’s contradictory stuff like ‘That doesn’t make any sense so let’s work it out’.

#### Risks

Participants felt it was important to only communicate counterintuitive information when the science was already counterintuitive: ‘You have to be careful with the temptation to be counterintuitive when it’s not quite there’. Communicators wanted to avoid ‘sensationalising’ the science and expressed risks when the science being communicated is not surprising: ‘The difficulty with that is biasing something because you are looking for a surprise result or prioritising stories that give you surprise results’.

Participants noted that it is important to communicate that science is normally quite intuitive: ‘Sometimes it’s important to tell the expected story. Sometimes it’s important to let people know that science and math can actually just follow the normal path, follow the expected path and that’s not necessarily a bad thing’. Communicators noted that counterintuitive results in science are less likely to be accurate than expected results and wanted to avoid the possibility that what they were communicating:

was just an unexpected result from one study: you have to be careful when you are thinking about counterintuitive storytelling, because you might step into pseudo-science or not validated science. Because science will be constructed over time it makes it less surprising.

Because science is often intuitive, one participant worried that making information counterintuitive could be perceived as manipulative: ‘it would have to be quite strategic and not make it feel like it’s something that manipulates people just for them to remember’.

Another participant noted that making something counterintuitive may run the risk of alienating an audience: By saying:

You think this, but actually here’s what’s really going on; it can have an effect on the audience in that they feel belittled, or the storyteller comes across as being very smug and all knowing; you don’t want to put people off following the information you’re trying to give them by telling them that they’re wrong about something.

Participants also noted that counterintuitive information may make things confusing:

things being counterintuitive can sometimes just then just become downright confusing. And I think at the end of the day, intuition is I think part of being able to engage with something; I think you’d have to present it quite carefully to make sure that the majority of the time was spent on the reveal rather than the inaccurate information so that people didn’t go away with a false impression of what you’re talking about.

### Considerations for negative information bias

#### Opportunities

Negative information was seen as a tool that can be used to engage an audience:

The lay audiences I have written for I try to add in some sadness without like dramatising someone’s grief or something like that. It does work. It’s brutal but humans are brutal, I guess; sad stories sell newspapers. The audience connects with it and they watch all that sadness. It has to be a really tremendous, fantastic, happy story to have the same impact.

Some science communicators saw negative information as having a lot of power: ‘negativity gets your attention, gives you something to think about, makes you feel like something important is happening’. Some communicators felt that negative emotions can lead to action:

If I was regularly engaging more in risk communication or climate change communication [. . .] there’s plenty of negatives there that could lead to good action; You need that negativity cause you need, to feel like ‘What can I do about it?’

One participant mentioned that this power to promote action depended on the audience: ‘I think positivity is really important for certain audiences and negativity is really important for others and it really depends what your action is at the end. Like doom and gloom equals action or positive hope equals action’.

Participants saw no issue with taking advantage of negative information if the content was already negative:

It would be wrong to say that the whole of science is about amazement and joy. There’s an awful lot of hard slog and disappointment [. . .] these negative emotions are in there. I think it’s also important to acknowledge them; I think there are places where negative might be helpful. For example, in health research, where you’re talking perhaps about the negative impact of cancer and things like that.

One interviewee felt that criticality went hand in hand with negativity, which is ‘important to have around science’.

Other participants observed that negative aspects in communication can help teach people, especially children, about failure and that ‘they don’t always have to win every time’. Participants noted that:

Research is failing 1000 times to succeed once, and letting people know that and then maybe they can internalise that scientists are doing a lot and they’re not just like, immediately have all the answers to everything; I want to try to change people’s understanding of who scientists are or what they’re like. And this is where this idea of saying, well, you know, not everything went well and it took me years to do this and I had a huge amount of failures along the way [. . .] I think it gives my audience permission to feel ‘Do you know what it didn’t work, but that’s alright.

One participant noted that the threat of failure may increase engagement: ‘There’s a possibility of things going wrong and audiences anticipate that and that can be a really strong engagement hook’.

#### Risks

Of all the cognitive biases, negative information was the most controversial, with all participants expressing concerns, saying it needed to be used ‘carefully’. Some resistance was simply about not wanting people to have any sort of negative experience or emotion: ‘I think if you were to fill your show with negative, that would be dreadful. You want positives. That’s what we’re after in life. We wanna have positive experiences’. Others emphasised not wanting the final emotion that they leave people with to be negative, wanting to avoid their communication being, in the words of one participant, ‘a bit of a downer’: ‘You wanna leave people often with some kind of optimism’; ‘It feels so often like you need to leave with some message of hope’; ‘We feel compelled to have a positive outcome to everything’. There was a feeling that if negative information was present, it needed to be accompanied by positive information: ‘I think that you can have more negative things in there if you have more positive things as well’.

Some participants acknowledged that negative information is likely to be more memorable, but they still try to avoid it: ‘If I see something kind of shocking or scary or worrying then you know that’s gonna stick with you more. And it’s just funny, though, because it’s kind of the opposite of what we try to do’. Some participants worried that making something negative might make people suspect it is untrue:

There might be some people that see the negative aspect of the information and think that therefore confirms it’s manufactured, it’s fear mongering. It’s, you know, ‘I don’t believe it as much because I can see that it’s designed to provoke a reaction and therefore I don’t think it’s true’ or something like that.

Participants wanted to avoid fear or anxiety:

We don’t wanna leave people with a sense of fatalism or gain a sense of panic as well. You know, we don’t wanna scare anyone; we try not to just kind of shock and scare, but actually have a more optimistic feel.

Participants expressed that fear can be debilitating and cause people to not act in situations where the danger feels too great:

You can’t make people anxious and depressed so much that they can’t do anything about it or can’t feel motivated or feel like it’s individually their fault;

you want to balance the seriousness of what’s happening and you don’t want to sugarcoat anything and you don’t want to lie to anyone but at the same time you don’t wanna leave them with that overwhelming sense of fatalism.

Or else create feelings of denial: ‘an overload of negative information could end up causing then a denial or a lack of wanting to engage’. Fear was also cited as something that might remove people’s confidence: ‘Negative things are there, but I think there’s a danger of them being scary and robbing people of confidence’.

Some worried that making information negative might alienate audiences or put them off science:

It feels so often like you need to leave with some message of hope because disaster communication in my experience tends to turn me off the subject because I just can’t deal with it; science to me, is already sort of somewhat unattainable or misunderstood. [. . .] I’d probably try and avoid [negative emotions] because I’m trying to not necessarily spin science in a positive light, but make people feel that it’s accessible to them and they can understand it and something they can partake in, or at least enjoy.

Participants also worried that making information negative would contradict some other objective they have with their communication: ‘They’re trying to either recruit or raise aspirations or raise sort of attitude and that’s hard to mesh with having people have those sort of negative experiences’.

More than one participant communicated a worry that making communications negative over a period of time could ‘desensitise’ people or make them ‘negatively fatigued’. One participant noted that the bias towards negative information may not work if we make everything negative: ‘if all you get is negative coverage, people tune out, like people tune out single valence stories pretty quickly, especially if there’s a lot of them’.

### Considerations for survival information bias

#### Opportunities

Survival information bias was seen as useful to science communication, especially in contexts of existential threat, such as the climate crisis, medical sciences and pandemics: ‘You can think about what can we do to help us counteract climate change or how should you behave during the pandemic? It’s all survival. And people engage with these kinds of news pieces’. Some communicators thought that a survival frame could work for any kind of science: ‘Relating things to people’s well-being is a really good opportunity and I think that can be done with any kind of science’. Although others expressed that it would very much depend on the type of science being communicated.

Participants saw survival as being a cognitive bias that can put a more positive frame on communication that includes negative information:

There is clearly a negative story there too, right? Because obviously there was some terrible thing that happened for someone to survive, but it’s paired with a positive story, which I think is really good. I can see that working well; I see it as a positive way of storytelling. I cannot envision immediately negative aspects of it because everything, it’s almost related to solution journalism, right? You can think about what can we do to help us counteract climate change or how should you behave during the pandemic? It’s all survival.

#### Risks

As with all cognitive biases, survival information can be hard to implement for some subjects: ‘There’s not that much within theoretical math that sort of does that like you usually need to go at least 2 degrees removed to get to something like that’. There was some worry that emphasising a survival frame may distract from the science: ‘No problem seeing a survival component, but really the core of the story isn’t about that. And so they can sort of lose the main message’, or may downplay narratives of things that don’t cause an existential crisis, but still need attention, such as medicine that can improve quality of life, rather than save lives. One participant noted that survival was a ‘minimum’, because they want people to do more than survive: ‘people would like to, if not thrive, at least exist in that space between just surviving and thriving and enjoying things’.

Some participants worried that survival information had the same risks as negative information: ‘Having people feel stressed or saddened or anxious [. . .] I suppose it impacts the experience that the visitors or people being engaged would have. And again their caretakers, if they would want [children] to be exposed to those feelings’. Participants worried that survival stories can involve trauma, so asking people to share survival stories may be difficult: ‘Survival stories for people usually involve quite heavy trauma, right? So asking people to share those, or having people that are willing to share those can be really difficult’. Or having audiences exposed to traumatic stories may require extra support: ‘they might need a chance to talk with somebody or decompress afterwards after this kind of narrative’.

There was some worry that scientists overcoming problems and surviving might contradict the message science communicators want to send about science:

I think sometimes we can portray scientists as superheroes. That they have done something that an everyday person can’t do and think that sometimes makes it hard for the audience to relate to because they feel that they’re not on the same level as these sorts of amazing superheroes in the stories.

Some felt that emphasising survival information might overemphasise the survival of some groups more than others, leading to issues for inclusive communication:

There could be a danger that we would lose diversity and inclusivity if we went down that road. It’s quite easy to frame it too tightly around a small group or some individuals who get the benefit and survive when actually we need always to think a bit broader than that.

One participant mentioned the need for the survival aspects to be relevant to the audience: ‘It has to be a timeline that makes sense to them and has some sort of urgency associated with it’.

### Themes

The inductive analysis highlighted several themes across different cognitive biases. These themes related to the objectives, topics or media of science communication.

### Science communication has a purpose

Many participants expressed that the objective of their storytelling was not merely to have audiences remember information. Science communicators wanted audiences to have fun, engage and raise aspirations. They wanted to recruit people to careers, increase participation and inclusivity and encourage behaviour change. They wanted to dispel misunderstanding, ensure people understand new information fully and change attitudes. Some expressed that audiences remembering and understanding information is important, but this wasn’t enough:

Memorability is great, but what does that memorability actually mean? If there’s no further on question of is that leading to things like behaviour change? Memorability isn’t enough what we’re doing; the kind of memory that we want is a memory that inspires action and positivity and change. And if the kind of memory that you’re referring to doesn’t translate into that, then that’s not necessarily the type of memory that we want to trigger.

This mismatch between what makes information memorable and some other objective was most prominent when participants were talking about the negative information bias: ‘The negative can be very memorable. But I wonder whether that always leads to action’. There was a worry that negative information can leave people ‘feeling like everything’s screwed and there’s nothing that can be done’ and does not ‘inspire action and positivity and change’ or ‘leave them feeling empowered and positive’, or else that we simply do not know the effects it might have: ‘You can make someone engaged in like gripped to a negative story but what is the effect of that on their consciousness? What is the effect of that on their behaviour?’

### Maintaining scientific integrity

Participants had a resistance to the use of cognitive biases where there was risk of misrepresentation of the science or a true story:

A fiction author can do anything. We’re slightly more bound by the facts have to be correct; You’ve got to be true to the science. If something is intuitive, then you don’t want to go out of the way to use counterintuitive content; you’re often trying to shoehorn in a narrative that isn’t actually there; [the cognitive bias] would have to be something that would naturally be there. There was a strong sense of wanting to maintain honesty in communication: You don’t want to sugarcoat anything, and you don’t want to lie to anyone; you’ve got to stay true to whatever it is that you’re talking about, so you can’t force something into a survival-oriented story if it isn’t related to what’s going on.

Participants did not want to sensationalise science:

You’d kind of threaten the integrity of your other projects if one was deemed to sort of be a bit over the top; I don’t think a science communicator would exaggerate for the sake of exaggeration. At least I hope not. Maybe that is a danger.

There was a fear sensationalised content might be associated with untrustworthy content: ‘There is this sort of fear or sort of desire to not make things sound sensationalist, to worry about being lumped in with sources that can’t be as trustworthy because they do rely on the sensationalism’. And some worried that stories that appeal to cognitive biases may be less likely to be robust:

You’re drawn towards stories that are less sort of replicable in some way. You’ll find the thing that’s like a person willing to say the thing that grabs attention. But is that the thing you should be highlighting if the intuitive story might be more realistic?

### Scientific topics can limit stories

Science communicators mentioned concerns for when cognitive biases were not directly relevant to the communication topic: ‘It’s sometimes difficult depending on the subject matter and you don’t wanna sort of force things in artificially’. This theme came out in relation to every cognitive bias. For instance, several communicators mentioned issues with making stories about survival when this was not relevant to the science. Communicators were generally happy to take advantage of a cognitive bias if the information was already present, but had concerns about including content that was not ‘naturally there’. However, there was some willingness to adapt content to appeal to certain biases. For example, reframing stories to centre human characters or making non-human stories have characters through anthropomorphisation:

If you can think of it a bit more abstract as like different things being people, and I think that works really well; there might not even be multiple participants unless you get into things like anthropomorphisation where it’s like the other character in the story is the centrifuge, you know, rather than another human being.

### Making communication simple

Some communicators worried that using some cognitive biases, such as counterintuitive and social information, might make communication more complicated:

If you would have to explain why it was counterintuitive, and why this was a surprise, and that might make the story telling a bit more complicated; I could see a problem with maybe that middle step of adding in something that’s a bit too complicated for someone to follow anything and when people can’t follow they kind of give up.

For some, complicating a story was about not wanting to confuse people, but for others it was about not wanting to detract from other aspects of the storytelling that were being prioritised: ‘They want that visually spectacular experience so really getting into the sort of nitty gritty of a character would be quite difficult’, or wanting to make the production of the story too complicated: ‘The more people you involve, the more complicated it gets because it’s more difficult to gather people together’.

### Protecting welfare of audiences

Communicators stressed the importance of looking after the welfare of audiences. They did not want to leave people with a negative emotion after engaging with science communication and wanted to protect audiences from feelings of anxiety, powerlessness or fatalism. Communicators also expressed concern for the welfare of people telling their stories and characters in stories who may be real people. Communicators did not want to tokenise trauma, use trauma to get engagement, or profit from stories of real trauma or grief:

Featuring stories of people that have been through a difficult experience, you just have to be quite careful about how much agency you’re giving them within the story. It’s something that you think about, am I kind of removing this agency and using this story as a dramatic device?; it seems like you are using those sad stories as your profits [. . .] I don’t feel comfortable if I have to do it and I don’t feel comfortable when I watch it or when I read it.

One participant specifically linked their duty of care for minoritised groups to stories which might have elements of trauma:

We’ve done things where we’ve spoken with indigenous groups and also artists on some of the basis for their works and those are deeply personal and traumatic. And when you have those explored in a public set spectrum, you have to also make sure that you support the people coming to these events.

## Discussion and conclusion

This research has explored whether and how science communicators take advantage of cognitive biases within their storytelling practice. Because of their embedded position within storytelling practice with science communication objectives, our participants were able to critically discuss the aspects of cognitive biases that create opportunities or pose risks for science communicators.

Science communicators reported already taking advantage of some cognitive biases in their practice. Communicators seemed happy to feature cognitive biases when the content they were communicating already lent itself to the cognitive bias. For instance, taking advantage of counterintuitive bias when the science was already surprising or social information when the science is already human-centred. Communicators also identified objectives within their communication that the cognitive biases might be useful for. For example, using social information to communicate about science as a team endeavour and using negative information to communicate about failure.

While some communicators expressed willingness to find ways to make their stories take advantage of biases, they also expressed some practical problems of implementing biases into their storytelling practice, including issues with resources, keeping stories short and suitable for certain media and the time it takes to craft stories. Communicators who collect stories from others (e.g. journalists) expressed specific practical challenges, due to not having control over the stories that they told. Some worried that using cognitive biases too much might desensitise audiences to these tactics. However, the literature cited here does not suggest that cognitive biases become weaker with exposure.

Participants acknowledged that memorability was important for science communication, but not more than other objectives. For some objectives, participants worried that cognitive biases might contradict the purpose of the communication. For example, communicators observed that some cognitive biases may prevent them achieving things like behavioural change or engagement if participants are made to feel fatalistic or anxious via negative information. Some participants acknowledged that anxiety, fear and caring about human suffering can be motivating, especially for spreading information and situations where individual action can be helpful, such as during the COVID pandemic. While some research is consistent with the belief that fear and anxiety are ineffective tools for motivating engagement with audiences (e.g. O’Neill and Nicholson-Cole, 2009), negative emotions (e.g. anxiety and anger) have been self-reported to be important in the emotional journey towards climate activism (Bright and Eames, 2022).

Some communicators felt cognitive biases could counter objectives by putting people off science if communication was too negative or alienating and belittling people through contradicting their beliefs or making stories too complex. There was a fear that making a story more counterintuitive or adding more characters could create a story too complicated or complex for certain audiences. This position might indicate a view of the audience as people who need information ‘dumbed down’ for them, an attitude typical of the deficit model of science communication, which views audiences as incapable of understanding much science content (Ko, 2016). Arguing against complexity in stories also contradicts the Social Brain Hypothesis (Dunbar, 1998), which argues that our brains evolved in order that we can deal with complex social problems. Indeed, we do every day in our daily lives as well as through the narratives we consume in films, TV and books. We may need to examine why some might feel that people can’t deal with these narratives in science communication.

Science communicators expressed a desire to protect the welfare of audiences, especially in relation to the cognitive biases for negative and survival information. Science communicators saw themselves as needing to protect audiences from feeling fear and anxiety, or from feeling alienated and excluded. Communicators wanted to keep communication honest, meaning that stories of grief, trauma and tragedy would need to be about real events. Some discussed discomfort with using real subjects with trauma as a tool to get engagement, to create profit or achieve any other objective, something that might be especially problematic when the communication isn’t helping the people whose story is being told.

A theme across cognitive biases was a worry that taking advantage of cognitive biases can make stories seem sensationalised. Some worried about the integrity of the science or story if it was changed to implement a cognitive bias. This concern may arise because of our participants, and science communicators more generally, having a background in science where integrity is of huge importance. Of course, we would not recommend using cognitive biases in ways that would alter the integrity of science, but further work could look at how cognitive biases affect the reception of scientific messaging. Some participants worried that cognitive biases might make stories look sensationalised or like misinformation, even when they remained honest and accurate to the science, making audiences more sceptical when they detect tactics often used in the spread of misinformation.

Collectively, constraints science communicators put on themselves to protect people, preserve integrity and honesty may put science communication on the back foot. While science communication can have relatively complex objectives and values, online actors only interested in gaining attention for advertising revenue can use cognitive biases uncritically, as well as those aiming to spread misinformation such as conspiracy theories, which are necessarily negative, socially orientated and counterintuitive (Cassam, 2023). While all science communicators have concerns about cognitive biases that may harm audiences, contradict objectives or compromise integrity, some go further in not wanting their communication to even look like they are using cognitive biases as it risks the perception that their communication lacks integrity or has welfare issues. In turn, avoiding cognitive biases may improve the believably of science communication. However, this analysis assumes a high level of media literacy around the tactics frequently used to spread misinformation.

The evidence and discussion here presents a rich and complex picture of storytelling practices in science communication. While it is hard to derive a message of best practice from the results, the discussion here highlights the importance of being aware of the empirical research and evidence around what makes stories stick with us if this is paired with critical engagement from science communicators, scrutinising these cognitive biases in relation to what we are trying to achieve in our science communication.

## Supplemental Material

sj-docx-1-pus-10.1177_09636625251387445 – Supplemental material for ‘A fiction author can do anything, we’re bound by the facts’: The risks and opportunities of taking advantage of cognitive biases in storytelling for science communication Supplemental material, sj-docx-1-pus-10.1177_09636625251387445 for ‘A fiction author can do anything, we’re bound by the facts’: The risks and opportunities of taking advantage of cognitive biases in storytelling for science communication by Hannah Little and Juliet Dunstone in Public Understanding of Science
