# Supplementary material for: ‘A fiction author can do anything, we’re bound by the facts’: The risks and opportunities of taking advantage of cognitive biases in storytelling for science communication
Source: Public Underst Sci. 2025 Nov 22;35(3):367–87. doi: 10.1177/09636625251387445 (PMC12999997; doi:10.1177/09636625251387445)
Supplement: sj-docx-1-pus-10.1177_09636625251387445 – Supplemental material for ‘A fiction author can do anything, we’re bound by the facts’: The risks and opportunities of taking advantage of cognitive biases in storytelling for science communication [file sj-docx-1-pus-10.1177_09636625251387445.docx]

**'A fiction author can do anything, we're bound by the facts': The risks and opportunities of using cognitive biases in storytelling for science communication**

**Supplemental Material**

**Hannah Little^1^ and Juliet Dunstone^1,2^**

^1^ The Department of Communication and Media, The University of Liverpool

^2^The Department of Psychology, The University of Stirling

**Contents**

1. Appendix 1: Questionnaire items
2. Appendix 2: List of codes
3. Appendix 3: Analysed Transcript Examples

## Appendix 1: Interview Questions and Script

1. What is your job?
2. How long have you worked in science communication?
3. Do you use storytelling in your science communication? If so, in what form do you tell stories?
4. Typically, who are the audience for your communication?
5. Do you have a qualification in science. If not, what is your academic background?
6. Have you had any formal training in science communication?

Preamble for cognitive bias questions:

“This study is all about storytelling and how, as science communicators, we can make our stories more memorable.

“Work in cultural evolution on folk tales and other forms of storytelling has demonstrated that there are aspects of stories that make them more or less memorable, and therefore successful as stories within an oral tradition.  
 
“In this research, we have identified 4 different features of stories that we know make them more memorable. We want to ask you about each of these features to establish how easy or useful it may be for you in your storytelling practice. We also want to ask about any potential issues these story features may be for your practice where there may be a conflict between framing a story in a particular way and the objectives you have for a story.

“If you’re unsure what I mean at any point, please let me know and I’ll give you an example.

1. Events in stories that involve more than one human (e.g. are social) make those stories more memorable than stories with one or fewer human characters. For instance, a story about humans affecting each other will be remembered more faithfully than some physical phenomenon affecting another physical phenomenon or a lone human.
2. Do you think knowing about this bias might be beneficial to your science communication practice in some way?

1. Do you foresee any potential problems with making your storytelling have  more social information in it?

Optional social example if asked for clarification: 
 
“For example, a story about how covid or some other disease affects an individual person will be less memorable than a story about how one human’s behaviour (e.g. social distancing, mask wearing) affects another person. “

1. Events in stories that are counter to our intuitive expectations also makes stories more memorable. For instance, if scientific results are surprising or unusual then they are more likely to be remembered.
2. Do you think knowing about this bias might be beneficial to your science communication practice in some way?
3. Do you foresee any potential problems with making events in your storytelling more counterintuitive?

Optional counterintuitive example if asked for clarification: 
 
“For example, a story about how vaccines don’t work will be more memorable than a story about how vaccines do work, because for most people previous experience leads to the expectation that vaccines will be effective.”

1. Events in stories that are that are negative for humans are more memorable than positive events. These events may be of greater importance to our survival than positive information because negative events enable us to avoid potential dangers, so emphasising the bad effects of not doing some behaviour may stick in people’s heads more than stressing potentially positive outcomes.
2. Do you think knowing about this bias might be beneficial to your science communication practice in some way?
3. Do you foresee any potential problems with making events in your storytelling more negative?

Optional negative bias example if asked for clarification: 
 
“For example, a story about how covid causes severe illness or death, or the negative impacts of climate change will be more memorable than stories about how vaccines prevent death, or how carbon consumption is improving. “

1. Events in stories that are that are survival-orientated are also more memorable, even when not negative. For instance, any information that may be useful for humans to survive a disease or other existential crisis.
2. Do you think knowing about this bias might be beneficial to your science communication practice in some way?
3. Do you foresee any potential problems with framing findings in your science communication to be more survival orientated?

Optional survival bias example if asked for clarification: 
 
For example, a story about the steps we can take to survive a pandemic will be more memorable than a story without this framing around survival.  
 
11. Do you have any other comments or thoughts about science communication and storytelling that we haven’t spoken about?

## Appendix 2: List of Codes

### Codes for Inductive Analysis

Benefits of Social Information

Risks of Social Information

Benefits of Counterintuitive Information

Risks of Counterintuitive Information

Benefits of Negative Information

Risks of Negative Information

Benefits of Survival Information

Risks of Survival Information

### Codes for Deductive Analysis

#### Science Communication has a Purpose

Bias increases engagement or attention

Bias detracts from an objective of scicomm

Bias detracts from communicating enough science

Bias prevents positive behaviour change

Bias prevents positive experience

Bias presents participation or inclusion

Bias can lead to compassion fatigue

Bias creates important learning moment

#### Maintaining Scientific Integrity

Bias increases risk of sensationalising information

Bias reduces scientific integrity

Bias reduces perceived scientific integrity

Bias risks reinforcing incorrect information

Honesty is important

#### Scientific Topics can Limit Stories

Bias is not sufficiently relevant to subject matter

Bias is relevant to subject matter

#### Making Communication Simple

Bias risks making information confusing or complex

Complexity more suited to longer-form media

Bias risks people don’t understand new information fully

#### Protecting Welfare of Audiences

Exploitation of contributors for purpose of scicomm

Ensuring grief or trauma was not tokenised or used for profit/engagement

Bias risks alienating audience or prevents inclusion

Bias risks upsetting audiences

Bias risks increasing climate anxiety

#### Practical Considerations

Lack of control when inviting others to tell their stories

Constraints of working for/representing an institution

Physical constraints on implementing bias

Stories easier in longer-form media

## Appendix 3: Analysed Transcript Examples

| Transcript example | Codes |
| --- | --- |
| Interviewer: Do you see foresee any potential problems of making the events in your science communication more counterintuitive?  Participant: I don't know whether it's directly relevant or not. It just reminded me of something that we cover in our science communication training that we do where and sometimes people do kind of these myth busting things and where I don't know, it might be like there's more ice growing in the Antarctic, you know, greater duration or extent of sea ice in the Antarctic.  And so some people might think that this is because it's getting colder down there, but actually it's because of all of these different climate processes.  But sometimes what can happen is that people in their busy lives not having a lot of time watch a bit of something, maybe even watch all of it.  But the thing that they only remember is the myth, and they don't actually remember the the counterpoint and that that they were really trying to hammer home.  Maybe some people haven't even, like, heard the myth, and then that introduces that into their head as a kind of seed and and so potentially something similar could happen in that people remember the counterintuitive or other, you know, the the opposite of of what it is, which I don't think is a huge problem.  But yeah, it just made me think of that.  And I think you'd have to present it quite carefully to make sure that the majority of the time was spent on sort of the the reveal rather than the inaccurate information so that people didn't who watched like, you know, the first part of the video, go away with a a false impression of of of what you're talking about.  I guess it's especially with things like social media where people kind of might start watching the video for 15 seconds and then go moving on and so not accidentally just feeding them as more piece of misinformation is something to be so careful of when people have short attention spans.  Yeah.  This is the problem.  Yeah.  And what sort of one we did our research and this is no disrespect to anybody, but sometimes sort of you you get people to watch a bit of something and then they'd have to write a short description of what the video is about.  And you think that wasn't what it was about at all?  Yeah.  And, you know, people kind of get the wrong end of the stick, and particularly if it's somebody you know outside of the field, not used to kind of interacting with that kind of content.  And as I said, I don't think it's a barrier so big that I'd never want to do.  I think it's a really cool idea and think you know, as I say, we've done similar things in the past and it's just I think you'd be careful in terms of  if it was on YouTube pick things like the title and the thumbnail to to make sure that you weren't just selling the, the preconceived notion and actually you were talking about like,  did you know that actually this is, this is the true case? | Unsure on use of bias  example  Practical - time  information confusing for audience  reinforcing incorrect information  information confusing for audience  practical - medium  reinforcing incorrect information  information confusing for audience  information confusing for audience  Desire to try bias  Tried bias before  Misrepresenting science  Honesty |

| Transcript example | Codes |
| --- | --- |
| Interviewer: Do you think that knowing about this social bias might be beneficial to your science communication in any way?  Well, it it means that I've been doing the right thing.  I so as as I said earlier like my focus does tend to be on the more human aspects, specifically in mathematics.  But what I'm doing when I'm talking about other sciences, I tried to stick to that as well.  And while often since I'm producing podcasts and doing a bunch of different interviews for the same story can be time consuming and sometimes just impossible to schedule, sometimes I will.  Oftentimes I will end up with one voice, but they're always talking about their interactions with other people.  Like I always make sure to ask those questions.  And so it is the sort of thing that is.  I mean, it's helpful to note that that does work.  So yeah, in in general like I I try to do this.  Especially when I'm talking to other people about mathematical communication.  Because so much so much in math removes the humanity, it removes the human, and it's so many math stories, which I mean are rare enough to see in the media.  Anyway, it's rare enough to see it. You get it a little bit better in the UK.  I have my personal opinions that it's mostly because of the Christmas lectures and the belief that people are allowed to talk about math, that that engenders, but even though it tends to be about a thing, right, like it doesn't tend to be about the people involved.  And I, uh, and that I think is a is a great tragedy.  And so, yeah, I'd like, this is something I tell nascent mathematical communicators to do so yes, I like I this is something is very important to me. | Tried bias before  Bias relevant to subject matter  Practical – time and media  Others telling their stories  Bias difficult for subject matter  Bias rarely used  Bias difficult for subject matter |
